# CrAssphage May Be Viable Markers of Contamination in Pristine and Contaminated River Water

**DOI:** 10.1128/msystems.01282-22

**Published:** 2023-02-06

**Authors:** Nyasha Mafumo, Oliver K. I. Bezuidt, Wouter le Roux, Thulani P. Makhalanyane

**Affiliations:** a DSI/NRF SARChI in Marine Microbiomics, Department of Biochemistry, Genetics and Microbiology, University of Pretoria, Pretoria, South Africa; b Water-Related Microbiology Laboratory, Water Centre, Pretoria, South Africa; UiT - The Arctic University of Norway

**Keywords:** bacteriophages, bacteria, crAssphage, metagenome assembled genomes, phylogeny, faecal pollution, viruses

## Abstract

Viruses are the most biologically abundant entities and may be ideal indicators of fecal pollutants in water. Anthropogenic activities have triggered drastic ecosystem changes in rivers, leading to substantial shifts in chemical and biological attributes. Here, we evaluate the viability of using the presence of crAssphage as indicators of fecal contamination in South African rivers. Shotgun analysis revealed diverse crAssphage viruses in these rivers, which are impacted by chemical and biological pollution. Overall, the diversity and relative abundances of these viruses was higher in contaminated sites compared to pristine locations. In contrast to fecal coliform counts, crAssphage sequences were detected in pristine rivers, supporting the assertion that the afore mentioned marker may be a more accurate indicator of fecal contamination. Our data demonstrate the presence of diverse putative hosts which includes members of the phyla *Bacteroidota, Pseudomonadota, Verrucomicrobiota*, and *Bacillota*. Phylogenetic analysis revealed novel subfamilies, suggesting that rivers potentially harbor distinct and uncharacterized clades of crAssphage. These data provide the first insights regarding the diversity, distribution, and functional roles of crAssphage in rivers. Taken together, the results support the potential application of crAssphage as viable markers for water quality monitoring.

**IMPORTANCE** Rivers support substantial populations and provide important ecosystem services. Despite the application of fecal coliform tests and other markers, we lack rapid and reproducible approaches for determining fecal contamination in rivers. Waterborne viral outbreaks have been reported even after fecal indicator bacteria (FIB) were suggested to be absent or below regulated levels of coliforms. This indicates a need to develop and apply improved indicators of pollutants in aquatic ecosystems. Here, we evaluate the viability of crAssphage as indicators of fecal contamination in two South African rivers. We assess the abundance, distribution, and diversity of these viruses in sites that had been predicted pristine or contaminated by FIB analysis. We show that crAssphage are ideal and sensitive markers for fecal contamination and describe novel clades of crAss-like phages. Known crAss-like subfamilies were unrepresented in our data, suggesting that the diversity of these viruses may reflect geographic locality and dependence.

## INTRODUCTION

The exposure to anthropogenic pollutants has resulted in the drastic decline in the quantity and quality of potable water. Aquatic pollutants include an assortment of toxic chemicals and pathogenic microorganisms (1, 2). In developing countries, threats to drinking water are exacerbated by the inadequate access to wastewater treatment facilities (3–5). As a result, drinking water sources may be exposed to human fecal contamination, which can result in low-quality drinking water, with increased potential of spreading waterborne diseases (6–9). Active surveillance to monitor and detect potential pathogens is vital for protecting public health and ensuring potable water (10–12). Currently, for microbial contaminants, these efforts have relied on the use of fecal indicator bacteria (FIB) such as Escherichia coli (5, 13, 14). However, the concentrations of FIB do not always correlate with the presence of some biological pollutants present in aquatic environments (15–20). This has been demonstrated by several studies, which have reported on waterborne viral outbreaks that occurred after analyses based on FIB were suggested to be absent or below regulated levels of coliforms (21, 22). This discrepancy demonstrates the failure of current methods and suggests the need to further develop new monitoring strategies, which account for all biological risks associated with contaminated water. This indicates a need for a universal marker, which is highly sensitive, for integration into modern microbial contamination surveillance protocols (23).

Viruses are the most biologically abundant entities on Earth and have been proposed to be better indicators of fecal pollutants (24–27). Several metagenomic studies have shown that crAssphage are the most abundant viruses in the human gut (28–31). The current data suggest that crAssphage cluster into five discrete groups designated as alpha-gamma, beta, delta, epsilon, and zeta (28), and span 10 distinct and phylogenetically diverse genera (32, 33). Based on the analysis of CRISPR spacer regions, and functional characterization, it appears that crAssphage mainly infect bacteria from the phylum Bacteroidota (28, 31, 32, 34, 35). Due to this abundance, these double-stranded (ds) DNA viruses may be ideal microbial source tracking (MST) markers of human fecal contamination (23, 36–38). Stachler and Bibby showed the potential utility of using crAssphage-based markers for tracking human fecal waste. The authors showed that crAssphage was highly host specific and highly abundant in sewage and biosols in Europe and the United States. The study also showed that crAssphage were detected in sewage samples from Asia and Africa, albeit at lower abundances. The results suggest that this phage is prevalent globally and support its application as a MST marker. Following these observations, several qPCR assays have been developed (37, 39). These assays have been successfully applied to quantify crAssphage in feces, wastewater, and surface waters in several regions, including parts of Europe (39–41), Asia (42, 43), North America (23, 44), South America, (45) and Australia (46). Evidence from these studies suggests that crAssphage may be highly specific for detecting human feces and sewage, with little or no cross-reactivity with animal feces, and hence, ideal MST markers. However, several studies have also demonstrated that crAssphage may not occur exclusively in the human gut, but may be present in the guts of animals and feces, albeit at lower concentrations (36, 47, 48). Nevertheless, there is an urgent need to investigate the suitability of using crAssphage as a biomarker of fecal contamination in underrepresented and understudied geographic locations such as Africa, to assess the feasibility of using the virus as a universal marker (48–50).

**FIG 1:**
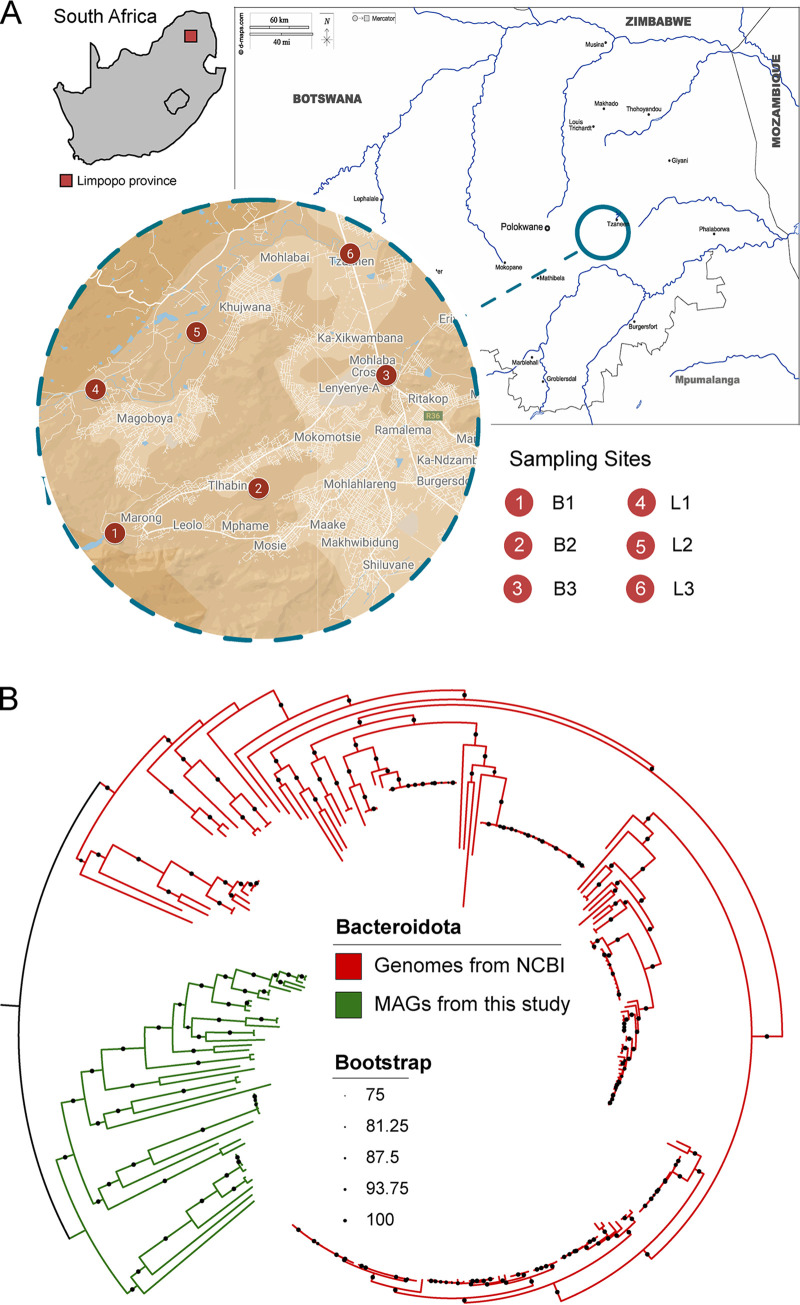
Map of sampling sites and a phylogenetic tree of Bacteroidota. (A) The six sampling sites in the Limpopo, Province of South Africa. The letters designate specific sampling locations as with L and B for Letsitele and Thabina (known locally as the Bathabina) River, respectively. Specific sites include the following: L1, upstream of the settlements and irrigation; L2, midstream; L3, downstream of the settlements; B1, upstream of the settlements; B2, midstream; B3, downstream of the settlements. (B) Bacteroidota maximum-likelihood phylogenetic tree. The metagenome assembled genomes (MAGs) obtained from this study are indicated in green. Those retrieved from the NCBI database are shown in red. The tree illustrates separate clustering between the MAGs obtained in this study and those from the NCBI. The genomes from this study form a separate and distinct cluster. Bootstrap values were calculated to support the robustness of the different clades and are indicated by the insert. The Limpopo map was sourced from dmaps (https://d-maps.com/pays.php?num_pay=1641&lang=en), and the inset was sourced from Google Maps.

To reduce this knowledge deficit, we used shotgun metagenomic analysis to explore the diversity of crAss-like phages in two South African rivers (Fig. 1A). Based on previous studies (36, 37, 40, 49), we predict that crAssphage in contaminated sites will be more abundant and diverse. We selected three conserved capsid and genome-packaging proteins (terminase large subunit, portal proteins, and major capsid proteins) as markers for detecting crAss-like phages in samples collected from both pristine and contaminated sites. We characterized and classified crAss-like sequences from these environments into subfamilies and identified distinct clades of diverse crAss-like phages.

## RESULTS

### The diversity of bacterial genomes in river systems.

In total, 297 bacterial metagenome assembled genomes (MAGs) were reconstructed from our six metagenomes. These MAGs constituted 115 high and 149 medium quality bins, which were dominated by *Pseudomonadota*, *Actinobacteriota*, and *Bacteroidota* phyla, respectively (Table S1). A phylogenetic reconstruction of *Bacteroidota* showed that MAGs from this study clustered separately from NCBI complete reference genomes (Fig. 1B). Furthermore, the data suggest a clear genetic discontinuity between our *Bacteroidota* and those from the NCBI, based on ANI scores ≤88% (Table S2). Similarity searches using 298 CRISPR spacers, detected in the high and medium quality MAGs, predicted 16 potential bacterial hosts. These hosts were associated with crAss-like viruses recovered from our samples. Among these 16 putative hosts, 14 belonged to *Bacteroidota*, and remaining hosts were affiliated with *Verrucomicrobiota* and *Pseudomonadota*. Further analysis, using iPHoP, resulted in the prediction of six *Bacteroidota* and two *Bacillota* putative hosts (Table S3). This expanded the known pool of putative hosts to three, and added increased the repertoire of phyla infected by our crAss-like viruses.

10.1128/msystems.01282-22.1 TABLE S1 The total number of metagenome assembled genomes (MAGs) recovered in this study, including information regarding the Bin ID, marker lineage, completeness, and contamination. Download Table S1, XLSX file, 0.02 MB. Copyright © 2023 Mafumo et al. 2023 Mafumo et al. https://creativecommons.org/licenses/by/4.0/ This content is distributed under the terms of the Creative Commons Attribution 4.0 International license.

10.1128/msystems.01282-22.2 TABLE S2 Information regarding the shared average nucleotide identity (ANI scores). Data comparing *Bacteroidota* from this study with those from the NCBI RefSeq database. Download Table S2, XLSX file, 0.01 MB. Copyright © 2023 Mafumo et al. 2023 Mafumo et al. https://creativecommons.org/licenses/by/4.0/ This content is distributed under the terms of the Creative Commons Attribution 4.0 International license.

10.1128/msystems.01282-22.3 TABLE S3 The crAss genomes and the putative hosts predicted by Crispr and iPHoP. Download Table S3, XLSX file, 0.01 MB. Copyright © 2023 Mafumo et al. 2023 Mafumo et al. https://creativecommons.org/licenses/by/4.0/ This content is distributed under the terms of the Creative Commons Attribution 4.0 International license.

### CrAssphage relative abundances in contaminated and pristine water.

**FIG 2:**
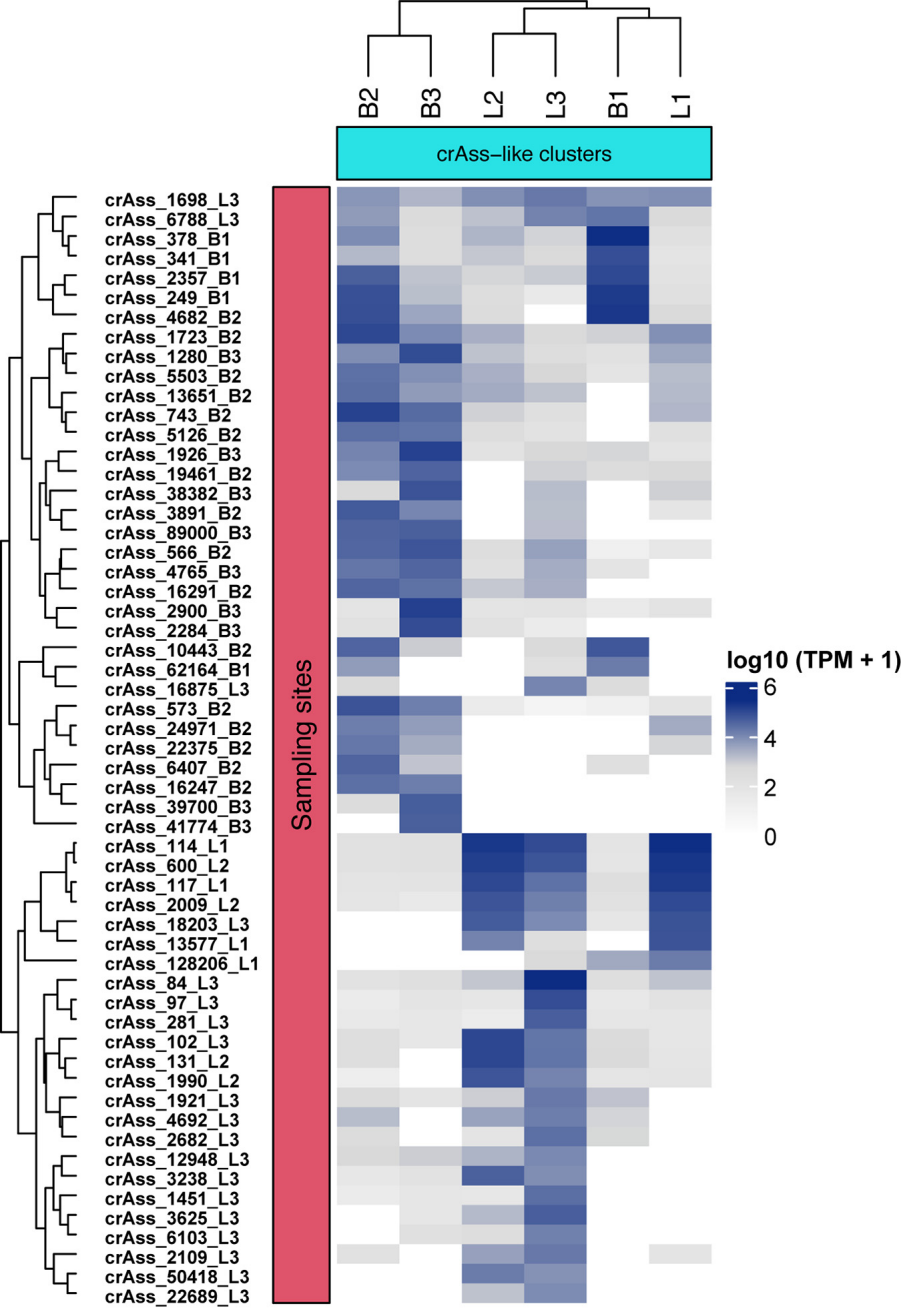
The relative abundances of crAssphage viruses. The relative abundance was calculated by read mapping sequences from the different sampling locations. The six sites (L1, L2, L3, B1, B2, B3) are shown in the *x* axis. The labels B and L correspond to Thabina and Letsitele river sampling sites, respectively. The cluster analysis was based on Euclidean distances. The putative crAssphage sequences, obtained from this study, are shown on the right *y* axis. The dark blue color indicates high crAssphage abundances, in a specific site, while white shows the absence of crAssphage per location. The heatmap shows generally higher diversity and distribution of crAssphage observed in contaminated sites compared with pristine sampling locations.

As a proxy for crAssphage relative abundance, transcripts per million (TPM) values (Table S4) were generated to evaluate the distribution and diversity of crAss like phages in the different sampling sites (Fig. 2). The relative abundances suggest that some crAss-like phages may be site specific. For instance, several crAss-like phage genomes (crAss 13577, crAss 22689, crAss 50418) were found in high abundances in samples from Letsitele but were absent in Thabina. Furthermore, we also identified specific crAssphage genomes (crAss 12948, crAss 16291, crAss 3238), which were completely absent upstream of settlements (sites B1 and L1). However, these genomes were identified in downstream sites, which were located within the vicinity of human settlements and are subject to human fecal contamination due to the use of pit latrines (sites B2, B3, L2, and L3) (Table S4). Overall, as predicted, we observed higher relative abundances and diversity of crAss-like phages in the contaminated sites (B2, B3, L2) compared to pristine sites (Fig. 2).

10.1128/msystems.01282-22.4 TABLE S4 The relative abundances of crAssphage obtained from the different sampling sites calculated using transcripts per million (TPM) values. Download Table S4, XLSX file, 0.01 MB. Copyright © 2023 Mafumo et al. 2023 Mafumo et al. https://creativecommons.org/licenses/by/4.0/ This content is distributed under the terms of the Creative Commons Attribution 4.0 International license.

### Diversity of uncharacterized crAssphage in rivers.

**FIG 3:**
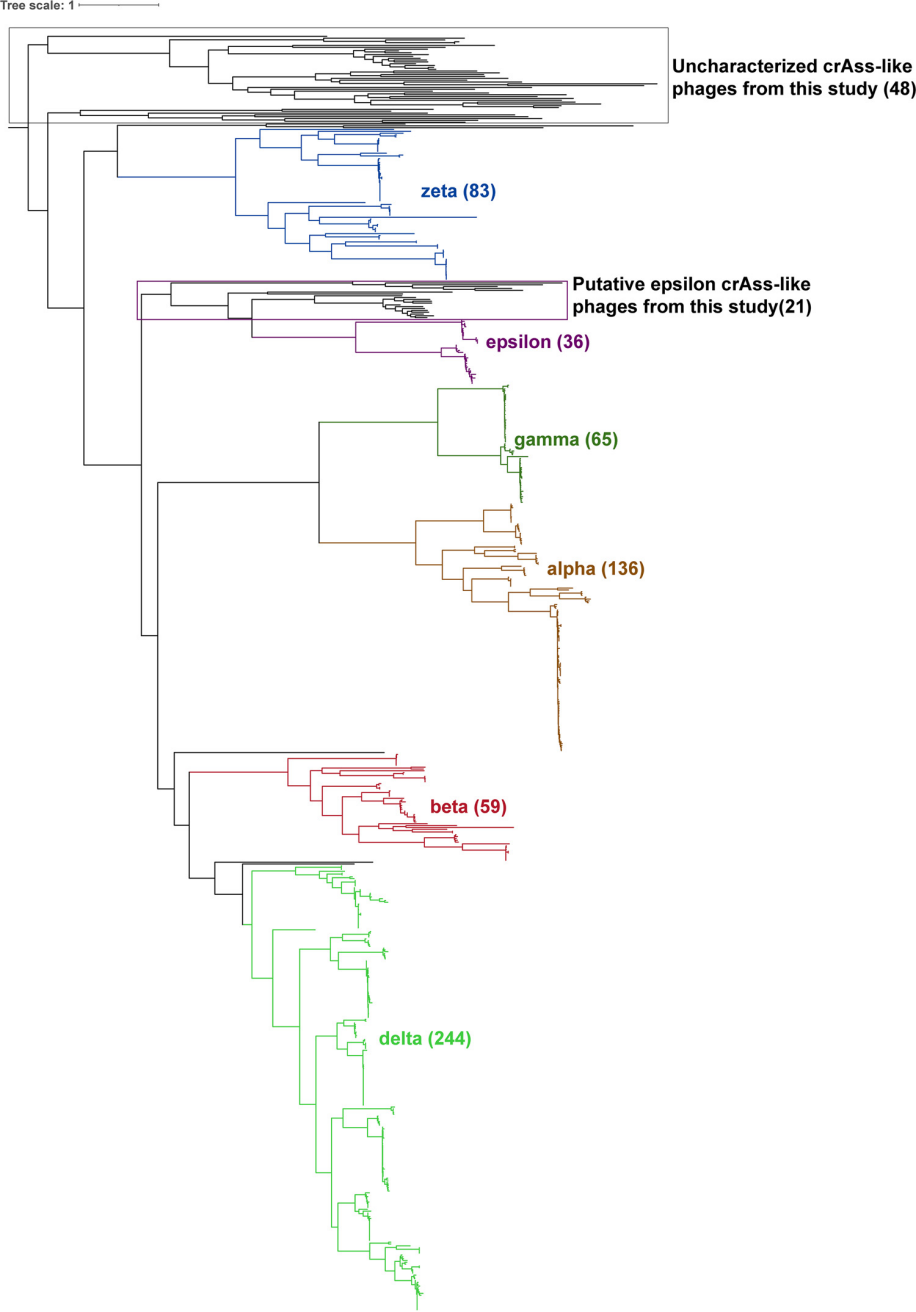
Phylogenetic tree of crAssphage sequences. The tree was constructed using TerL protein sequences from crAssphage. CrAssphage, obtained from this study, are colored black. CrAssphage sequences obtained from a recent study (28) representing Zeta, Epsilon, Gamma, Alpha, Beta, and Delta clusters are shown in blue, green, purple, brown, red, and green, respectively. CrAssphage obtained in this study clustered with the Zeta, Epsilon, and Delta subfamilies. In general, crAssphage retrieved from this study clustered separately from those obtained from the Yutin et al. (28) study. This suggests that they may potentially represent novel, and as yet, uncharacterized crAssphage sequences.

HMM profile searches, using major capsids, terminase large subunits (TerL), and portal proteins, resulted in the prediction of 384 crAss-like contigs across all metagenomes. Of the overall predicted crAss-like contigs, only 57 harbored ≥2 hallmark genes, representing a total of 50 vOTUs. Of these, we found six high-quality viral contigs, five medium-quality, and 46 low-quality viral contigs due to the highly fragmented nature of these sequences (Table S5). Moreover, the reconstruction of phylogeny, using dereplicated TerL protein sequences (51), showed the diversity of crAss-like phages associated with our data (Fig. 3). From this analysis, we observed that around 64% (48 of 75) of our crAss-like TerL sequences clustered separately from the previously proposed groups, whereas the remaining (34) sequences clustered with known Epsilon and Delta subfamilies.

10.1128/msystems.01282-22.5 TABLE S5 Summary statistics showing the quality of crAss contigs with two or more hallmark genes obtained through quality assignments with CheckV. Download Table S5, XLSX file, 0.01 MB. Copyright © 2023 Mafumo et al. 2023 Mafumo et al. https://creativecommons.org/licenses/by/4.0/ This content is distributed under the terms of the Creative Commons Attribution 4.0 International license.

**FIG 4:**
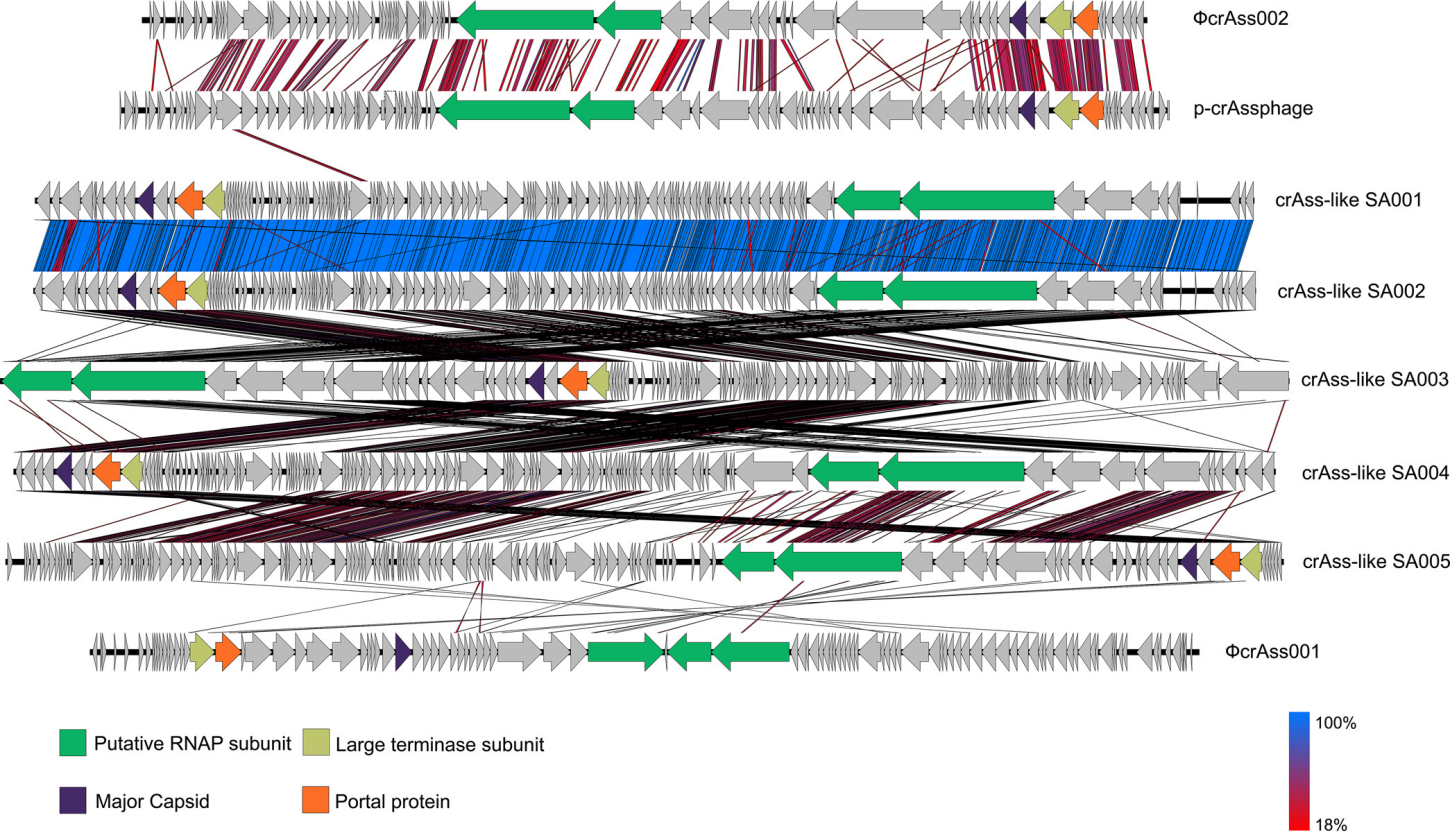
Genomic structure comparison of near complete Epsilon crAssphage. The crAssphage sequences from this study were compared with the prototypical crAssphage (p-crAssphage), and two other gut associated crAssphage. Regions of amino acid sequence homology are shown. Blue indicates 100% homology and red indicates the lowest similarities. The RNAP subunit and the capsid gene module were found in all the genomes analyzed.

Of the total crAss-like phage predictions, 20 contigs had all three hallmark genes. The size of the contigs ranged from 12 to 119.3 kbp. Of these, 17 contigs were classified as putative Epsilon (16) and Delta (1) subfamilies, respectively, based phylogenetic placements using TerL proteins. The Epsilon crAss-like phages were the largest contigs (Data set S1). We selected five high-quality and near-complete contigs, with size ≥100 kbp, and compared these to three human gut associated crAss-like genomes (Fig. 4). The analyses suggest that crAss-like contigs from our data were highly similar in comparison to reference genomes. However, the order of genes related to the RNAP subunit and to the capsid gene module, was conserved across all genomes (Fig. 4).

## DISCUSSION

CrAssphage are potentially ideal MST markers for detecting human fecal contamination due to their abundance, specificity, and sensitivity (36, 37, 52–54). However, few studies have investigated the viability of using crAssphage as potential markers in environmental water at both global and local scales (36, 48, 50). In this study, we conducted *in silico* analysis to determine the feasibility of crAssphage as indicators of water contamination in two South African rivers. The sampling sites in the rivers were designated as pristine or contaminated based on fecal quality assessments (Table S6). Our analysis suggests that consistent with our prediction, crAssphage appear to be more abundant in contaminated sites (B2, B3, and L2) compared to pristine sites. We also reveal highly diverse and uncharacterized crAssphage in these rivers, expanding the genomic repertoire of known crAss-like clades.

10.1128/msystems.01282-22.6 TABLE S6 Information regarding contamination levels and the sampling locations. The total coliform counts (MPN/100 mL) and the E. coli counts (MPN/100 mL) obtained from the different sites. The GPS coordinates are shown alongside the coliform counts. Download Table S6, XLSX file, 0.01 MB. Copyright © 2023 Mafumo et al. 2023 Mafumo et al. https://creativecommons.org/licenses/by/4.0/ This content is distributed under the terms of the Creative Commons Attribution 4.0 International license.

By expanding the known crAss-like clade, our data shed new light regarding the established potential hosts of these viruses. Previous studies suggest that crAssphage primarily infect Bacteriodata (28, 31, 32, 34, 35). Host prediction using two independent approaches suggests the presence of three other potential host phyla, *Bacillota*, *Verrucomicrobia*, and *Pseudomonadota*. These results corroborate a recent *in silico* prediction by N. Yutin et al. (28), which proposed *Pseudomonadota* and *Bacillota* as other putative hosts of crAssphage. These findings provide additional evidence confirming that crAssphage may have a wider range of hosts than initially predicted. In addition to the expanded host phyla, phylogenetic analysis suggests that the *Bacteriodata* from our study may be more diverse than previously described genomes. This is perhaps unsurprising as the majority of current *Bacteroidota* genomes were isolated from human guts of western populations (55–57). Our MAGs were retrieved from African rivers, which are under different evolutionary selective pressures (e.g., temperature, physicochemical variables) compared to gut bacteria (58). It has been established that differences in geographical origin, ethnicity, and urbanization substantially shape the diversity of microbiota (59–61). It is possible that these variables may contribute to the disparities observed in this study at the species level (60). Our findings suggest that crAssphage host diversity may be substantially under characterized. It is possible that, depending on the environmental niche, crAssphage may associate with other hosts. For instance, Verrucomicrobia, which are known to be more abundant in soils (62, 63), may be more ideal hosts for crAssphage in these niches.

The relative abundance estimates suggest that crAssphage dominate impacted river sites. This result validates our proposal that these sequences may be viable markers of contamination and is consistent with previous studies based on quantitative PCR (37–40, 42–44). A previous study by Stachler et al. used sequence data to demonstrate the potential of crAssphage based biomarkers. To the best of our knowledge, this study provides the first evidence, based on genome resolved metagenomics, showing the applicability of crAssphage sequences as markers of contamination in South African rivers. While FIB were useful in providing a general indication of pristine and contaminated sites, our analysis suggests that crAssphage may be more sensitive markers. In addition to their sensitivity (38, 43, 54, 64), previous studies suggest that crAssphage are more resistant to environmental stress compared to FIB (65). The ability to withstand environmental stress may favor the use of these indicators as ideal markers of contamination.

An additional benefit of using crAssphage, as molecular markers of contamination, is their diversity and global distribution (28, 32, 66, 67). Based on evaluating the diversity and phylogeny of crAss-like viruses, the 48 TerL protein sequences from our data were highly distinct relative to known clades. This distinct clustering suggests that crAss-like phages from our data set may represent highly diverse subfamilies, reflects geographic locality and dependence (48), and further supports our assertion regarding evolutionary niche selection of these bacteriophages. Previous work on wastewater has provided strong evidence of geographic dependence of crAssphage (36, 48). These studies hypothesized that the abundances and diversity of crAssphage would be higher in European and U.S. samples, compared to those in Africa and Asia (36, 48, 66). Similar to findings on host microbiota, the variation in the diversity of crAssphage may be explained by the differences in urbanization and diet (59, 68, 69). These differences have been shown to drive changes in both host and phage genomes (66, 69). A small proportion of the remaining TerL sequences (27) identified in this study were classified with known Epsilon, Delta, and Zeta subfamilies. Epsilon sequences were recently described by N. Yutin et al. (28) and appear to be dominated by sequences from gut microbiota. The Delta subfamilies constitute the largest group of gut crAss virome and are known to be distantly related to the Epsilon subfamilies (28). The identification of sequences from these subfamilies hints at the presence of human fecal contamination in the river samples. However, relative to the other sequences, these subfamilies were underrepresented in the sampling area. The majority of these were affiliated with uncharacterized crAss-like sequences, reported in this study, representing a potentially novel clade. This finding further supports the effects of selective pressures and crAssphage niche specificity.

To further elucidate the diversity of crAssphage, comparison of the genomic structure of five near-complete sequences retrieved from this study was done. By comparing these sequences with ФcrAss001 (35), ФcrAss002 (70), and prototypical crAssphage (p-crAssphage) (31), the analysis revealed high variability in similarity and gene order conservation among the phages. From the five epsilon crAssphage, two were nearly identical, and three were highly homologous. This finding supports the assertion that sequences from this study were both novel and distinct from previously characterized phages. The observed differences suggest that, although the five crAss-like phages were classified within the epsilon subfamily, these may in fact represent four new genera (32, 33) and calls for the revision and possible expansion of the current taxonomy. Together these results support our proposed view regarding the potentially diverse assortment of uncharacterized crAss-like sequences in various environments.

### Conclusion and future prospects.

Our findings increase the repertoire of known crAss-like phages. The expanded taxonomy, including potentially novel clades, establishes a baseline for the identification of as yet unknown environmental crAssphage. These novel clades reported in this study were linked to new host phyla (*Bacillota*, *Verrucomicrobia*, and *Pseudomonadota*), confirming previous reports which demonstrated that crAssphage are not exclusively associated with *Bacteroidota*. In addition, our analysis supports the use of crAssphage viruses as biomarkers of fecal contamination. Using crAssphage as microbial source tracking markers may assist in mitigating the spread of waterborne diseases due to their robustness and sensitivity. However, future studies in environmental crAssphage viruses are required to validate these observations across a variety of river systems. These validations are required to establish the diversity of these sequences, which may result in the application of crAssphage as quantitative markers of fecal contamination.

## MATERIALS AND METHODS

### Study area, sample collection, and processing.

The samples were collected in the Limpopo Province of South Africa, in the Bathlabile tribal area, following consent from the Bathlabile Traditional Council. The area relies on water sourced from the Thabina and Letsitele rivers, which are subject to chemical and biological pollutants. The chemical pollutants in the Thabina river are primarily due to the application of organic fertilizers to nearby citrus farms, while biological pollutants in both rivers are in the form of fecal matter from humans (due to the use of pit latrines) and free roaming animals.

Six water samples were collected from pristine and polluted river waters. The selected locations were distributed at three sites along the Thabina and Lestsitele rivers (Fig. 1A). The B1 and L1 sites being from a pristine source, upstream of settlements, and agricultural practices. The B2 and L2 sites were in the middle of settlements, whereas B3 and L3 sites were downstream of the settlements (Fig. 1A). Five liters of water were collected from each location for metagenomic analysis and stored on ice. An additional liter of water from each sampling location was collected for standard water quality analysis at the Council of Scientific and Industrial Research (Table S6). The pH, turbidity, coliform, and E. coli counts were determined for each sample (Table S6). Each sample was filtered through a 0.2 μm polycarbonate (PES) filter membranes (Merck, RSA). DNA extractions from these filters followed, using the Qiagen Power Soil DNA Isolation Kits (Qiagen, USA) according to the manufacturer’s instructions. The quality of the resultant DNA was evaluated using gel electrophoresis (1% agarose) and its concentration was determined with Qubit dsDNA assay kit in Qubit 4 Fluorometer (Thermo Fisher Scientific, USA). High-quality DNA from each sample was sent for shotgun sequencing using an Illumina MiSeq (2 × 150 bp) at Admera Health (NJ, USA).

### Metagenomic analysis.

To elucidate potential crAssphage hosts, we generated MAGs from shotgun data. Raw paired-end metagenomic reads (2 × 150 bp) were quality trimmed, assembled, and binned using the ATLAS pipeline version 2.4.4 with the qc, assembly, and binning parameters (51). Briefly, using default versions of the tools below, the reads were quality filtered using BBTools version 37.99 (https://jgi.doe.gov/data-and-tools/bbtools/), assembled using metaSPAdes version 3.13.1 (71), and MAGs were generated using both metabat2 (72) and maxbin2 (73). The resultant bins were combined, refined and dereplicated using DAS_Tool version 1.1.2 (74) and dREP version 3.0.0 (75). The dereplicated MAGs were assessed for quality and completeness using CheckM version 1.1.5 (76). Using previously defined standards, MAGs with completeness >90% and contamination <5% were classified as high-quality (Table S7) and those with completeness of ≥50% and contamination of <10% were classified as medium-quality (77) (Tables S8 to 10).

10.1128/msystems.01282-22.7 TABLE S7 High-quality bins recovered from samples collected along the two rivers showing information regarding the bin ID, marker lineage, completeness, and contamination. Download Table S7, XLSX file, 0.01 MB. Copyright © 2023 Mafumo et al. 2023 Mafumo et al. https://creativecommons.org/licenses/by/4.0/ This content is distributed under the terms of the Creative Commons Attribution 4.0 International license.

10.1128/msystems.01282-22.8 TABLE S8 The medium-quality bins recovered from samples collected along the two rivers showing information regarding the bin ID, marker lineage, completeness, and contamination. Download Table S8, XLSX file, 0.01 MB. Copyright © 2023 Mafumo et al. 2023 Mafumo et al. https://creativecommons.org/licenses/by/4.0/ This content is distributed under the terms of the Creative Commons Attribution 4.0 International license.

### Taxonomic annotation of bacterial MAGs and host detection.

The taxonomy of all medium- and high-quality MAGs were inferred using the Genome Taxonomy Database Toolkit version 1.6.0 (78). Of these, we reconstructed a maximum likelihood phylogenetic tree using MAGs classified as Bacteroidota as these are primary hosts for crAssphage using GTOtree version 1.6.11 (79). We used single copy gene sets (80) specific for the *Bacteroidota* phylum. This tree comprised of 47 MAGs, generated from this study, and included 450 reference *Bacteroidota* complete genomes acquired from the NCBI RefSeq release 205 database (81). The phylogenetic tree was visualized and annotated using iTol version 6.6 (82). Following this, we conducted pairwise comparisons between the Bacteroidota MAGs from this study and the 450 reference Bacteroidota. For these comparisons, we established the criteria of shared average nucleotide identity (ANI) using FastANI version 1.32 (83).

### Hidden Markov models-based detection of crAssphage.

For the detection of putative crAssphage, we acquired 81,246 crAss-related protein sequences from both the NCBI RefSeq (81) and a recent study by N. Yutin et al. (28). These sequences were clustered into a nonredundant set of 20,039 protein sequences using CD-HIT version 4.8.1 (84) with parameters: -c 0.9 -n 5 -aS 0.8. The representative sequences from these clusters were further compared for shared similarities using BLASTp (80) with e-value 1e-05. The blast results were clustered using Markov Clustering algorithm (MCL) version 14.137 (85) with 1.5 inflation. Clusters associated with major capsids, terminase large subunits (TerL), and portal proteins were individually aligned using MAFFT version 7.487 (86) with the -auto parameter, and the resultant alignments were converted to hidden Markov models (HMM) profiles using hmmbuild version 3.3.0 (87). To search for putative crAss-like viruses in our data, contigs were predicted for open reading frames (ORFs), using Prodigal v2.6.3 (88), with the -a and -p meta parameters. The profiles were subsequently searched against all protein sequences predicted in our contigs using the -T 50 parameter in hmmscan version 3.3.0 (87). Contigs predicted to be in possession of ≥2 of these crAss-like hallmark genes were clustered based on 95% sequence identity with over 80% of the shortest contig resulting in 50 viral OTUs and further assessed for quality using CheckV version 0.9.0 (Table S5).

### CrAssphage relative abundance and host detection.

As a proxy for determining the distribution and relative abundances of crAss-like phages across sampling sites, the CoverM version 0.6.1 (https://github.com/wwood/CoverM) tool was used to calculate TPM values. We used 57 crAss-like contigs with ≥2 hallmark genes and parent metagenomic reads. The resultant relative abundance estimates were then visualized using ggplot2 (89) in R v3.6.0 (90). Furthermore, these crAss-like contigs were also processed for host detection. MAGs generated from this study were searched for CRISPR spacers using MINCED version 0.4.2 (https://github.com/ctSkennerton/minced/tree/master). The identified spacers were probed for shared sequence similarity against the 57 crAss-like contigs, using BLASTn with the following parameters: -e-value 0.01 -word_size 8 -dust no -perc_identity 90. The crAss-like contigs were analyzed for host detection, using the iPHoP (integrated Phage Host Predictions) version 1.1.0 (https://bitbucket.org/srouxjgi/iphop). The taxonomies of our MAGs were first reclassified with GTDB-Tk version 2.1.0 using the de_novo_wf parameter as required by the pipeline and were further integrated into the database of hosts. Following this, the iPHoP pipeline was run using the default parameters.

### Genome comparisons and subfamily classification of crAss-like phages.

To determine the crAss-like subfamilies in our data set, we retrieved 96 complete TerL protein sequences from all our metagenomes. Protein sequences were clustered based on 95% sequence identity, over 80% of the shortest contig, resulting in the representation of 75 viral OTUs. These were supplemented, and subsequently aligned with 623 other TerL sequences acquired from N. Yutin et al. (28) using MAFFT version 7.487 (86) with the -auto parameter. The resultant alignment file was used to reconstruct a maximum likelihood tree with 1,000 bootstraps using IQ-TREE version 1.6.6 (91). The tree was then midpoint-rooted, visualized, and annotated using iToL (82). This was followed by comparisons of the five high-quality near complete (≥100 kbp) epsilon crAss-like genomes against each other, as well as ФcrAss001, (35), ФcrAss002 (70) (two pure culture isolates), and a prototypical crAssphage (*in silico* derived) (31) to assess relatedness using EasyFig version 2.2.2 (92) using tblastx with parameters: e-value cut-off 0.001 and length filter 30.

### Data availability.

The metagenomic data have been deposited at NCBI under BioProject ID PRJNA894350. Metagenomic assembled genomes are available from https://doi.org/10.6084/m9.figshare.21640316. The bash script and HMM profiles used to search for crAss-like viruses in our data can be accessed from https://github.com/SAmicrobiomes/crAssZA.

10.1128/msystems.01282-22.9 TABLE S9 The genome assembly statistics showing summary statistics. This includes the number of contigs at different lengths, total lengths, GC content, N50, and L50 values from the six metagenomes. Download Table S9, XLSX file, 0.01 MB. Copyright © 2023 Mafumo et al. 2023 Mafumo et al. https://creativecommons.org/licenses/by/4.0/ This content is distributed under the terms of the Creative Commons Attribution 4.0 International license.

10.1128/msystems.01282-22.10 TABLE S10 Data showing the sequencing efforts from the six metagenomes, including statistics such as the total raw read counts, deduplicated reads, and the total high-quality reads from the different sampling sites. Download Table S10, XLSX file, 0.01 MB. Copyright © 2023 Mafumo et al. 2023 Mafumo et al. https://creativecommons.org/licenses/by/4.0/ This content is distributed under the terms of the Creative Commons Attribution 4.0 International license.
